# Do’s and Don’ts in Primary Aneurysmal Bone Cysts of the Proximal Femur in Children and Adolescents: Retrospective Multicenter EPOS Study of 79 Patients

**DOI:** 10.1097/BPO.0000000000002267

**Published:** 2022-09-14

**Authors:** Thomas P.G. van Geloven, Lizz van der Heijden, Minna K. Laitinen, Domenico A. Campanacci, Kevin Döring, Dietmar Dammerer, Ismail T. Badr, Mikko Haara, Giovanni Beltrami, Tanja Kraus, Philipp Scheider, Camilo Soto-Montoya, Masood Umer, Marta Fiocco, Valentino Coppa, Pieter B. de Witte, Michiel A.J. van de Sande

**Affiliations:** * Department of Orthopedic Surgery; ## Department of Biomedical Data Science, Medical Statistics Section, Leiden University Medical Center; *** Mathematical Institute, Leiden University, Leiden, The Netherlands; † Bone Tumor Unit, Orthopedics and Traumatology; ** Department of Pediatric Surgery and Orthopedics, New Children’s Hospital Helsinki, University of Helsinki and Helsinki University Hospital, Helsinki, Finland; ‡ Department of Orthopedic Oncology and Reconstructive Surgery, Azienda Ospedaliero Universitaria Careggi; †† Department of Pediatric Orthopedics, Azienda Ospedaliero Universitaria Meyer, Florence; ††† Clinic of Adult and Paediatric Orthopaedics, Ospedali Riuniti di Ancona—Ospedale Pediatrico Salesi, Ancona, Italy; § Division of Orthopedics, Department of Orthopedics and Trauma Surgery; §§ Department of Trauma Surgery, University Clinic of Orthopaedics and Trauma Surgery, Medical University of Vienna, Vienna; ∥ Department of Orthopedic Surgery, Medical University of Innsbruck, Innsbruck; ¶ Department of Orthopaedics and Traumatology, University Hospital of Krems, Krems; ‡‡ Pediatric Orthopedic Unit, Orthopedics and Traumatology, Graz, Austria; # Department of Orthopedic Surgery, Menoufia University, Shebin El-Kom, Menoufia, Egypt; ∥∥ Department of Orthopedic Surgery, Instituto Nacional de Cancerologia, Bogotá, Colombia; ¶¶ Department of Orthopedic Surgery, Aga Khan University Hospital, Karachi, Pakistan

**Keywords:** ABC, pediatric, benign bone tumor, hip, survival, percutaneous treatment, weight, bearing

## Abstract

Aneurysmal bone cysts (ABC) are rare benign cystic bone tumors, generally diagnosed in children and adolescents. Proximal femoral ABCs may require specific treatment strategies because of an increased pathologic fracture risk. As few reports are published on ABCs, specifically for this localization, consensus regarding optimal treatment is lacking. We present a large retrospective study on the treatment of pediatric proximal femoral ABCs.

**Methods:**

All eligible pediatric patients with proximal femoral ABC were included, from 11 tertiary referral centers for musculo-skeletal oncology (2000-2021). Patient demographics, diagnostics, treatments, and complications were evaluated. Index procedures were categorized as percutaneous/open procedures and osteosynthesis alone. Primary outcomes were: time until full weight-bearing and failure-free survival. Failure was defined as open procedure after primary surgery, >3 percutaneous procedures, recurrence, and/or fracture. Risk factors for failure were evaluated.

**Results:**

Seventy-nine patients with ABC were included [mean age, 10.2 (±SD4.0) y, n=56 male]. The median follow-up was 5.1 years (interquartile ranges=2.5 to 8.8).

Index procedure was percutaneous procedure (n=22), open procedure (n=35), or osteosynthesis alone (n=22). The median time until full weight-bearing was 13 weeks [95% confidence interval (CI)=7.9-18.1] for open procedures, 9 weeks (95% CI=1.4-16.6) for percutaneous, and 6 weeks (95% CI=4.3-7.7) for osteosynthesis alone (*P*=0.1). Failure rates were 41%, 43%, and 36%, respectively. Overall, 2 and 5-year failure-free survival was 69.6% (95% CI=59.2-80.0) and 54.5% (95% CI=41.6-67.4), respectively. Risk factors associated with failure were age younger than 10 years [hazard ratios (HR)=2.9, 95% CI=1.4-5.8], cyst volume >55 cm^3^ (HR=1.7, 95% CI=0.8-2.5), and fracture at diagnosis (HR=1.4, 95% CI=0.7-3.3).

**Conclusions:**

As both open and percutaneous procedures along with osteosynthesis alone seem viable treatment options in this weight-bearing location, optimal treatment for proximal femoral ABCs remains unclear. The aim of the treatment was to achieve local cyst control while minimizing complications and ensuring that children can continue their normal activities as soon as possible. A personalized balance should be maintained between undertreatment, with potentially higher risks of pathologic fractures, prolonged periods of partial weight-bearing, or recurrences, versus overtreatment with large surgical procedures, and associated risks.

**Level of Evidence:**

Level IV, therapeutic study.

Primary aneurysmal bone cysts (ABC) are rare benign cystic bone tumors mostly diagnosed in children and adolescents, which can be locally aggressive.^1^,^2^ ABCs contain multiple blood-filled cystic spaces divided by fibrous septa.^1^ Mean age at diagnosis for ABC is 13 years,^3^ with 75% to 90% diagnosed before the age of 20.^4^ About 2% of all benign bone tumors are an ABC,^5^ with an incidence of 0.14 per 100.000 individuals.^6^ ABCs are generally diagnosed because of complaints of pain, a palpable mass, or a pathologic fracture.^2^ Localization in the proximal femur is known for increased recurrence rates 7 and increased fracture risk.^8^,^9^ Six to 9% of ABCs are localized in the proximal femur.^7^,^10^ There is no consensus on the treatment of ABCs in this location.

The generally preferred method for diagnosing ABC is magnetic resonance imaging (MRI), often combined with histologic confirmation.^2^–^4^,^11^ Both are advised as telangiectatic osteosarcoma has similar imaging characteristics and therefore, this differential diagnosis should be ruled out.^1^,^4^

The goal of this therapy was to achieve local control with healing and remodeling of the lesion and surrounding bone while maintaining function and minimalizing complications like fractures, growth disturbances, and recurrences.^12^ Current treatment strategies for ABC include (sometimes after a period of watchful waiting): percutaneous intralesional sclerotherapy, for example, with polidocanol (Fig. 1), bone marrow injections, cryotherapy, decompression, curopsy (ie, biopsy and curettage), curettage with or without filling and/or chemical adjuvants and/or high-speed burring (Fig. 2), systemic therapies, radiofrequency ablation, embolization, percutaneous injectable bone substitutes, wide resections, and if deemed necessary, a wide range of osteosynthesis.^2^–^4^,^13^–^17^ Treatment strategies depend on location, size, symptoms, impending and present pathologic fractures, local considerations, and individual preferences. Because of this heterogeneity, there is no consensus or guideline.^2^,^3^ Currently, the most common surgical treatment for ABC is curettage, with or without filling.^2^,^11^ However, there is a growing interest in less invasive therapies,^12^ because of potentially faster functional recovery and less surgical-site morbidity. This would lead to less absence from school and sports activities, meaning that children can continue their normal activities, which are of substantial importance to their development.^18^ Nonetheless, this might be at the cost of higher recurrent or persistent cyst rates.

**FIGURE 1:**
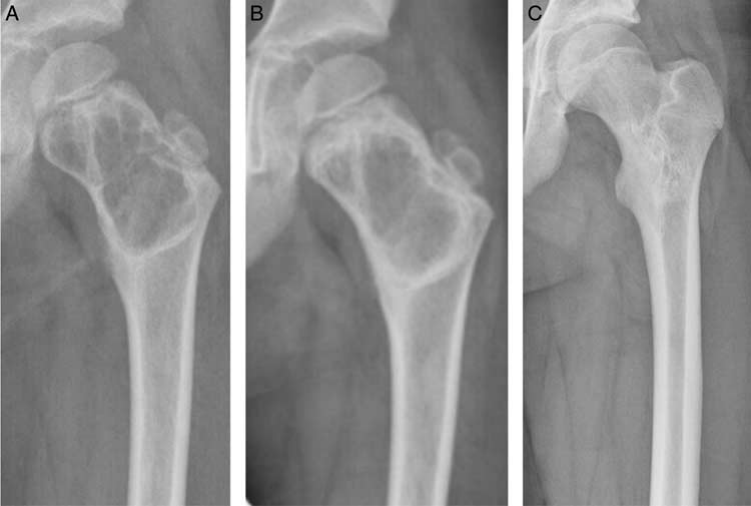
(A) Seven-year-old boy with an ABC in the left proximal femur treated with ethoxysclerol. (B) 3 months after first ethoxysclerol injection the intracameral septa had largely disappeared and the previously well-defined borders became vaguer. Patient received 3 more injections of ethoxysclerol over a period of 5.5 years, due to local recurrences. (C) After 5 years of follow-up, consolidation of the cyst and complete remodulation of the femoral neck and intertrochanteric region was observed, which was complete after 8 years.

**FIGURE 2:**
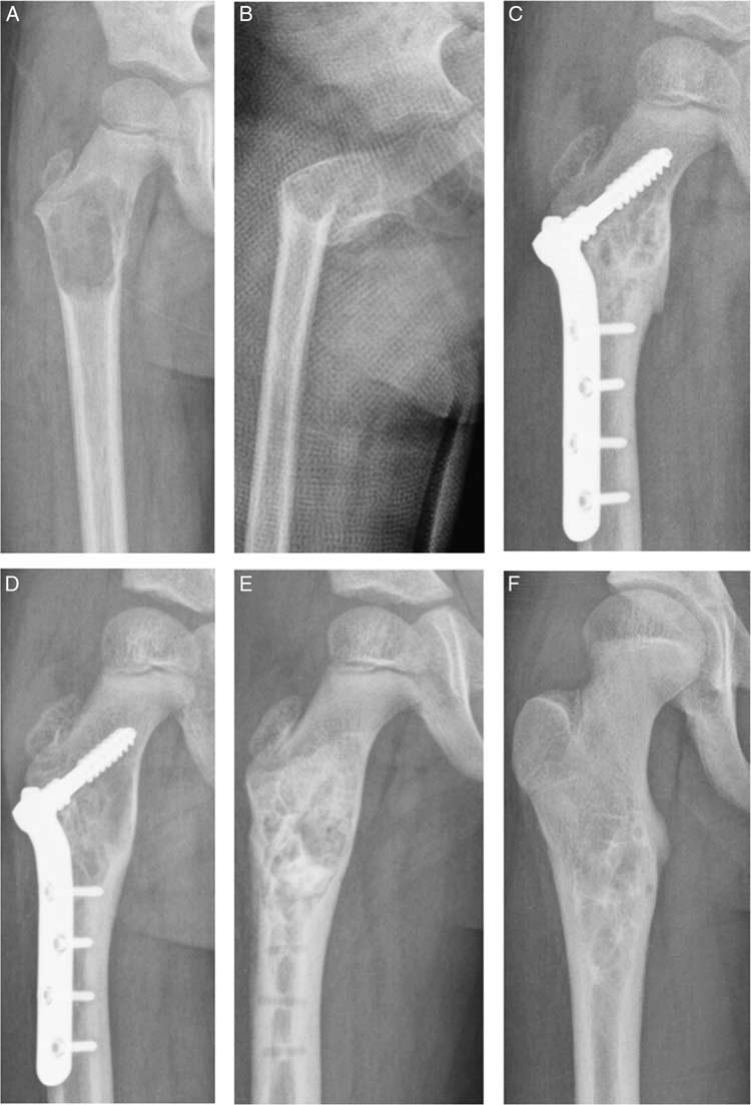
(A) Five-year-old boy with an ABC in the right proximal femur (B) Between diagnosis and initial treatment, a pathological fracture occurred. (C) Six months after curettage, with bone grafting, ethanol and a Coventry infant hip screw with a 4-hole plate. (D) Local recurrence of the ABC was seen 1 year after initial treatment (E) After plate removal, repeated curettage, bone grafting and ethibloc was performed (F) Complete filling and remodulation was seen after 3 years follow-up.

There is limited evidence on ABC treatment of the proximal femur, with mainly case reports, case series,^9^,^19^–^21^ and small retrospective studies on benign proximal femoral lesions.^11^,^22^,^23^ In these articles, the only reported treatments are curettage and osteosynthesis. Additional data is needed for tailoring surgical choices to specific ABC characteristics. The current study might help surgeons in decision-making for ABC treatment of weight-bearing bones. We have presented a large retrospective international multicenter study on ABC in the proximal femur, with the follow-up outcomes of a broad range of treatments, including curettage and osteosynthesis, as well as percutaneous procedures.

The aim of this multicenter study on proximal femoral ABCs in children was to evaluate treatment outcomes and failure-free survival (FFS). Further, we aimed to assess whether less invasive treatments can be applied in selected cases. The focus lies on lesion characteristics, success rates of various treatment methods, time until full weight-bearing, recurrences, and complications.

## METHODS

This multicenter study was initiated by insert center and promoted and joined by members of the European Paediatric Orthopaedic Society (EPOS), the European Musculo-Skeletal Oncology Society (EMSOS), and the International Society of Limb Salvage (ISOLS). All eligible pediatric patients with proximal femoral ABCs, treated in 1 of the 11 participating international tertiary referral centers for musculo-skeletal oncology between 2000 and 2021 were included (Table 1).

**TABLE 1:** Age at Diagnosis and Follow-up is Given in Years, Width and Distance to Physis in mm, volume in cm^3^.

Patient Characteristics	Percutaneous Procedure	Open Procedure	Osteosynthesis Alone	Total Group
Group size	22	35	22	79
Demographics				
Male, n (%)	13 (59)	27 (77)	16 (73)	56 (71)
Age at diagnosis (y), mean (SD)	9.9 (4.2)	9.8 (4.0)	11.4 (3.6)	10.2 (4.0)
Follow-up (y), median (IQR)	4.5 (2.7-9.3)	4.6 (2.3-9)	4.1 (2.6-7.6)	5.1 (2.5-8.8)
Localization				
Epiphysis, n (%)	1 (4.5)	6 (17.1)	0	7 (8.9)
Metaphysis, n (%)	6 (27.3)	19 (54.3)	15 (68.2)	40 (50.6)
Meta-diaphysis, n (%)	15 (68.2)	9 (25.7)	7 (31.8)	31 (39.2)
Diaphysis, n (%)	0	1 (2.9)	0	1 (1.3)
Size				
Width (mm), median (IQR)	37 (29-60)	38 (31-45)	50 (40-63)	41.1 (31.0-60.0)
Estimated volume (cm^3^), median (IQR)	54 (30-112)	64 (35-88)	51 (32-90)	54.6 (34.3-89.0)
Closest distance to physis (mm), median (IQR)	21 (7-35)	20 (8-49)	8 (0-33)	17 (6-42)
Fracture at diagnosis, n (%)	5 (23)	12 (34)	10 (45)	27 (34)

According to Dutch law and our Institutional Review Board, this retrospective study was not subject to the Medical Research Involving Human Subjects Act (G19-064) and therefore needed no informed consent. Data were collected by individual centers from medical charts and entered into a pseudo-anonymized database. All patients aged younger than 16, with a primary ABC localized in the proximal femur were included. The diagnosis had to be confirmed by MRI and/or histology and minimal postoperative follow-up was 6 months. Patients with involvement distal to the isthmus of the femur, due to the different surgical challenges these lesions represent, as well as cysts with prior treatment elsewhere (eg, failure or cyst recurrence at presentation), or comorbidities increasing fracture risk, like rickets, osteogenesis imperfecta, or enchondromatosis, were excluded.

Demographics, diagnostic features, and treatment characteristics were evaluated, as well as complications and reinterventions during follow-up. Cyst volume was approximated using maximal APxCCxML (mm).

Index procedures were categorized as percutaneous treatment (eg, sclerotherapy, decompression, radiofrequency ablation, and/or filling with injectable bone substitutes); open surgical treatment (eg, curettage, with or without adjuvants, filling, or osteosynthesis); or osteosynthesis alone (ie, without specific additional treatment of the ABC).

Outcome measures were 2 and 5-year FFS and time until allowed full weight-bearing as index procedure. In addition, a number of recurrences, time until recurrence as index procedure, and the number of complications were evaluated. Failure was defined as fracture during follow-up, the need for more than 3 percutaneous interventions, open surgical intervention, or recurrence, for percutaneous treatment.^12^ For open surgical treatment and osteosynthesis alone, failure was defined as fracture during follow-up, surgical reinterventions, or recurrence/persistent cysts. Recurrences were defined as cyst progression or recurrence after the index procedure.

### Statistical Analysis

Descriptive analyses were performed on baseline data for the complete group and stratified for index procedure in tables and a flow chart. Continuous data were described using means and SD or medians and interquartile ranges (IQR) in case of skewed distribution. Categorical variables were summarized as the number of observations and percentages (%).

Comparisons between treatment groups were made using 1-way analysis of variance for age at diagnosis, and with Kruskal-Wallis for follow-up, cyst width, cyst volume, distance to physis, and time until the first recurrence. Comparisons between treatment groups were made using χ^2^ test for sex, location, pathologic fracture at diagnosis, recurrences, and if failure occurred.

FFS from the index procedure was estimated by using Kaplan-Meier methodology. The cumulative incidence of time until allowed full weight-bearing was estimated by 1-minus Kaplan-Meier. The log-rank test was used to assess the difference between the survival outcomes.

To study the association between risk factors and survival outcomes, univariate Cox-regression models were estimated. Covariates used were sex, age at diagnosis under 10 years, fracture at diagnosis, and volume over 55 cm^3^. These prognostic factors were chosen on the basis of previous literature, with higher recurrence rates reported for younger children, males, and patients with pathologic fractures,^24^–^27^ higher failure rates in young patients,^12^ and increased fracture risk for larger lesions in the proximal femur.^11^ The cut-off point of age less than10 years has been frequently used for recurrence.^25^–^27^ Exact volume has not been described, so median volume was used in our study. Estimated hazard ratios (HR), along with their 95% confidence interval (CI) were reported.

No imputation methods were used on missing data. IBM Statistical Package for Social Statistics (SPSS) version 25 (Chicago, IL) was used for analysis.

## RESULTS

Of 85 pediatric and adolescent patients with ABC in the proximal femur, 79 were included (Table 1). Four patients were excluded because they were referred with local recurrence and prior primary treatment elsewhere. Two patients were excluded due to insufficient follow-up (2 and 4 mo).

Overall, the mean age at diagnosis was 10.2 years (±SD=4.0) and 71% (n=56) were males. Overall median follow-up was 5.1 years (IQR=2.5 to 8.8), only 6 patients had a follow-up of <1 year: 2 patients had a follow-up of 7 months, 2 of 9 months, and 2 of 11 months. The diagnosis was confirmed by MRI in 71 patients (90%). In all 8 patients were without MRI, the diagnosis was confirmed with histology. Overall, 49 biopsies (62%) were performed to confirm the diagnosis.

**FIGURE 3:**
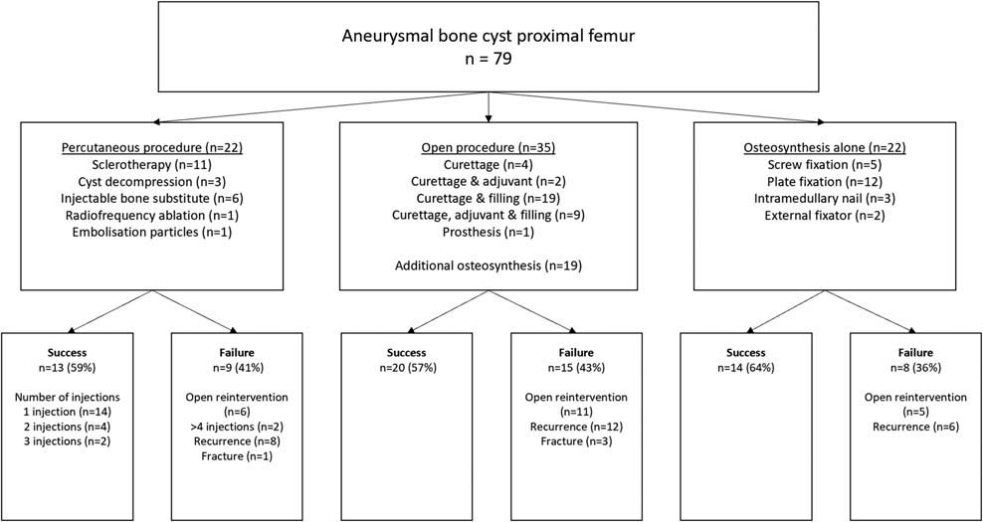
Flowchart of index procedures and their successes and failures.

Index procedures were percutaneous treatment (n=22), open surgery (n=35) (of which 19 with additional osteosynthesis), or osteosynthesis alone (n=22) (Table 2; Fig. 3). Two patients (aged 5 and 12) were observed with watchful waiting (8.6 and 6 mo) before receiving their index procedure (sclerotherapy and plate-osteosynthesis, respectively). During this period, 1 patient had a fracture resulting in a time until full weight-bearing of 21 weeks.

**TABLE 2:** Index Procedures

	Aneurysmal Bone Cyst (n=79), n (%)
Percutaneous procedure	22 (27.8)
Sclerotherapy	11 (13.9)
Decompression	3 (3.8)
Injectable bone substitute (HA)	6 (7.6)
Radiofrequency ablation	1 (1.3)
Embolization with particles	1 (1.3)
Open procedure	35 (44.3)
Curettage	4 (5.1)
Curettage and adjuvants	2 (2.5)
Curettage and filling	19 (24.1)
Curettage and adjuvants and filling	9 (11.4)
Total hip arthroplasty	1 (1.3)
Additional osteosynthesis	19 (24.1)
Osteosynthesis alone	22 (27.8)
Plate	12 (15.2)
Screw	5 (6.3)
Nail	3 (3.8)
External fixator	2 (2.5)
Other additional procedures in same setting	
Hardware removal	5 (6.3)
Scar resection	1 (1.3)
Embolization paired with sclerotherapy	1 (1.3)
Other	1 (1.3)
Missing	13 (16.5)
No. open reprocedures in follow-up	
0	56 (70.9)
1	12 (15.2)
2	6 (7.6)
3 or more	5 (6.3)
Complications	
Infection	0
Fracture	4 (5.1)
Recurrence	26 (33.0)

Recurrences occurred in 26 patients (33%) during follow-up, after the following index procedures: percutaneous procedures 8/22 (36%), open procedures 12/39 (34%), and osteosynthesis alone 6/22 (27%) (*P*=0.57). The median time until the diagnosis of recurrence was 25 (IQR=4 to 26) months for percutaneous procedures, 14 (IQR=4 to 26) months for open procedures, and 24 (IQR=9 to 41) months for osteosynthesis alone (*P*=0.6).

Complications included fracture (n=4), femoral head osteonecrosis (n=2), femoral head deformity (n=1), femoral neck shortening (n=1), and pain, for which plate removal was indicated (n=1). Overall, 4 patients (5%) required total hip arthroplasty. Two patients due to recurrence leading to fracture (aged 5 and 14), 1 patient due to femoral head necrosis (aged 14), and 1 patient with pathologic fracture at diagnosis immediately as index procedure (aged 13).

**FIGURE 4:**
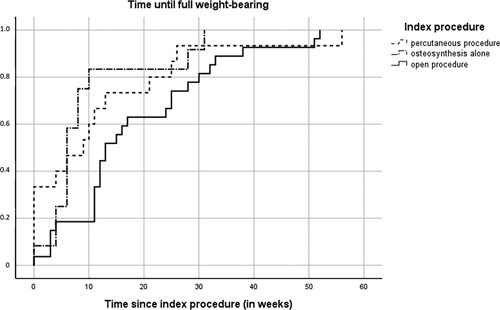
cumulative incidence of time until full weight-bearing.

Overall, the median time until allowed full weight-bearing was 11 weeks (95% CI=9.2-12.8): for percutaneous procedures 9 weeks (95% CI=1.4-16.6), for open procedures 13 weeks (95% CI=7.9-18.1), and for osteosynthesis alone 6 weeks (95% CI=4.3-7.7) (Fig. 4).

**FIGURE 5:**
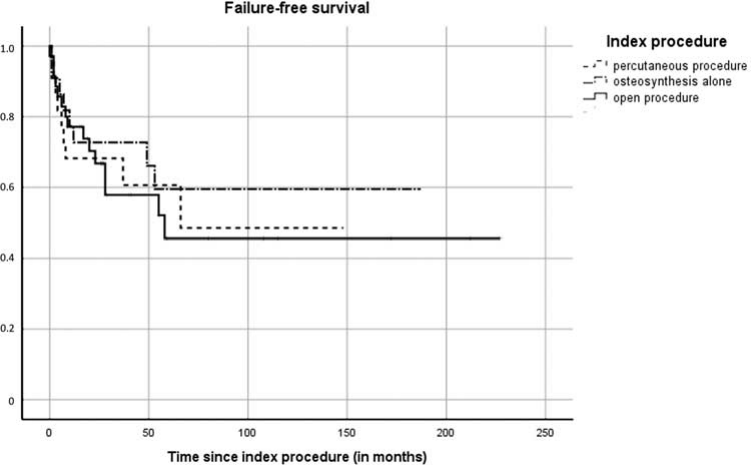
Kaplan-Meier curve of the failure-free survival.

Failure rates were: percutaneous procedures 41%, open procedures 43%, and osteosynthesis alone 36% (*P*=0.82; Fig. 3). Overall, 2 and 5-years FFS was 69.6% (95% CI=59.2-80.0) and 54.5% (95% CI=41.6-67.4), respectively. For percutaneous procedures, 2 and 5-years FFS was 68.2% (95% CI=48.8-87.6) and 60.6% (95% CI=38.3-82.9), for open procedures 66.8% (95% CI=50.5-83.1) and 45.6% (95% CI=24.6-66.6), and for osteosynthesis alone 72.7% (95% CI=54.1-91.3) and 59.5% (95% CI=37.0-82.4), respectively (*P*=0.75) (Fig. 5).

HR for failure: age younger than10 years (HR=2.9, 95% CI=1.4-5.8, *P*=0.003), volume >55 cm^3^ (HR=1.7, 95% CI=0.8-2.5, *P*=0.20), pathologic fracture at diagnosis (HR=1.4, 95% CI=0.7-3.3, *P*=0.26), and being male (HR=0.94, 95% CI=0.5-2.0, *P*=0.87) (Table 3).

**TABLE 3:** Potential Individual Risk Factors for Failure, Univariate Cox Regression Analysis

	N (Total 79)	Failure (Total 33)	Hazard Ratio	95% Confidence Interval	*P*
Age at diagnosis <10	31	20	2.9	1.4-5.8	0.003
Volume >55 cm^3^	37	12	1.7	0.8-2.5	0.20
Fracture at diagnosis	27	14	1.4	0.7-3.3	0.26
Male	56	23	0.9	0.5-2.0	0.87

## DISCUSSION

The aim of this multicenter study on ABCs in the proximal femur in children was to evaluate the outcomes and FFS of various treatment methods. We further aimed to assess whether less invasive treatments can be indicated for ABC in the proximal femur. We focused on lesion characteristics, success rates of various treatment methods, time until full weight-bearing, recurrences, and complications.

No significant differences in FFS survival between percutaneous treatment, open procedures, and osteosynthesis alone were found. The different lesion characteristics, however, result in accompanying risks for failure and are likely to have influenced the choice of treatment by orthopaedic surgeons. This might induce confounding by indication, but also reflects common clinical practice in the participating centers.

Younger age (<10 y) at diagnosis was a risk factor for failure in this series. Mohaidat et al,^25^ Gibbs et al,^26^ and Freiberg et al^27^ also found associations between younger age and recurrence. Dormans et al^10^ on the contrary, did not found significant links between age and recurrence/persistence rates. Because of the weight-bearing nature of the proximal femur, the risk for open reprocedures due to (impending) fractures is increased compared with other locations like the humerus,^8^,^9^especially in younger children with higher bone growth activity, who may be less susceptible for partial or nonweight-bearing instructions for fracture prevention. A Possible association between failure and volume >55 cm^3^ and fracture at diagnosis were found, however, without statistical significance. These factors influence the biomechanical stability of the proximal femur, resulting in fractures or the need for open re-operation, explaining increased failure rates in patients with these characteristics.

Time until full weight-bearing showed no statistically significant differences between treatment groups in our analyses, with 13 weeks for patients with open procedures, versus 9 weeks for percutaneous procedures and 6 weeks for osteosynthesis alone, although this result might be biased due to low power. A longer limited weight-bearing period after curettage might be explained by cortical structure weakening due to curettage, which could lead to surgeons choosing more apprehensive postoperative protocols. The existing literature on proximal femoral ABCs reports full weight-bearing for open procedures after 12 to 24-weeks.^9^,^19^,^20^ However, for percutaneous procedures or osteosynthesis alone in proximal femoral ABCs, full weight-bearing has not been described. In the latter case, we hypothesized that in analogy with simple bone cyst treatment, osteosynthesis that penetrate the cyst wall (screws, intramedullary nails, etc.) might cause the same spontaneous healing, as can be seen after biopsy or curopsy or injection therapy.

The full mechanism of healing in these instances was unknown to us, but we observed reasonably good results from osteosynthesis alone in the anecdotal cases series included in the article.

Our overall recurrence rate of 33% was similar to the 27.5% of Ramírez et al^28^ Our number may however be a small under-representation as recurrences might have occurred after the recorded follow-up in some patients. However, higher rates up to 71%^27^ and lower rates of 18% to 20%^7^,^10^,^29^ have been reported. Increased recurrence rates have been described for proximal femur localization of ABCs,^7^ as well as an increased fracture risk.^8^,^9^ Furthermore, juxtaphyseal localization or epiphyseal involvement, open growth-plates, and young age at diagnosis were identified in existing literature as risk factors for recurrences.^10^,^12^,^26^,^30^ In addition, Döring et al^31^ hypothesized anatomic locations to be a reason for differences in reported recurrence rates. These factors and the fact that we had a solely pediatric population, of which most still have open physes and an inherently increased recurrence risk, might explain the relatively high recurrence rate.

Four pathologic fractures were observed after the index procedure, 3 in patients with open procedures including osteosynthesis and 1 after percutaneous treatment (cement injection). This is remarkable, as over half of the study the population did not received osteosynthesis at index procedure and this only resulted in 1 fracture, indicating that not all pediatric patients with proximal femoral ABC have a risk of fracture in need of osteosynthesis, as often described in the literature.^9^,^11^,^19^–^21^,^23^ However, after a fracture, as was the case in 1 watchful waiting patient, ABCs very rarely show spontaneous healing, meaning further treatment is advised in these cases.^32^

In 2 patients, an initial watchful waiting policy was started before eventually requiring surgical treatment of their ABC. Because of the active nature of ABCs, watchful waiting is not advocated as definitive management; but the timing of treatment may be decided by patients and their parents with consideration of age, school, and sports schedules.

Where percutaneous procedures are often used for ABCs in proximal humerus,^12^ open surgery is often preferred for proximal femoral ABCs.^9^,^11^ In our data, percutaneous procedure outcomes seem comparable to those of curettage in the proximal femur. Possibly, percutaneous procedures are most suitable for specific cases, for example, smaller lesions and/or without impending fracture. In cases of no success after single or multiple injections, one can choose a more invasive technique such as curettage with or without osteosynthesis. In case of (impending) fracture where fixation is indicated, one could consider osteosynthesis alone, or curettage and filling combined with osteosynthesis.

The strengths of our study were the relatively large population, the inclusion of all conventional treatment options, and reporting of time until full weight-bearing. To the authors’ knowledge, this is the largest study on pediatric ABC in the proximal femur and the only study including both percutaneous injections and watchful waiting beside the more commonly described open procedures. Evaluating time to full weight-bearing has not been used before to describe treatment results of proximal femoral ABC, but is deemed an especially useful parameter in the outpatient clinic for pediatric patients and parents to better inform them on the expected impact of proposed treatments.

The limitations of this study were mainly confounding by indication, the lack of specific diagnostic criteria for all participating centers during the long study period, and the small subgroup size. The cyst size, location, and fracture risk, all influence the choice of index procedure by treating orthopaedic surgeons. Because of small group sizes, we could not correct this in a multivariate regression model. In addition, the retrospective and multicentered design of this study result in heterogeneous treatment methods, in concordance with the lack of consensus and the large role of personal preferences of treating physicians. On top of this, the multicentered design may have led to intercenter differences in diagnosing ABCs. A large number of specific treatment methods had to categorized into the aforementioned index-procedure groups for analyses. However, we believe that we have been able to roughly describe the result of these main treatment options despite these challenges.

For the treatment of proximal femoral ABCs in children and adolescents, failure rates of different treatments were comparable. Age younger than10 years at diagnosis seemed to increase the risk for failure. Because of the active and locally aggressive nature of ABCs, watchful waiting is not recommended as a treatment. Percutaneous treatment and open procedures are good treatment options in this weight-bearing localization, possibly reinforced with hardware in case of (impending) fracture. The aim of treatment should be maintaining local cyst control, with minimal complications, and ensuring children can continue their normal activities as soon as possible. A personalized balance should be maintained between undertreatment, with potentially higher risks of pathologic fractures or recurrences compared with overtreatment with larger surgical procedures and associated risks and a longer time to return to full weight-bearing.
